# The Effect of Family Factors on Intense Alcohol Use among European Adolescents: A Multilevel Analysis

**DOI:** 10.1155/2013/250215

**Published:** 2013-03-03

**Authors:** Kristjan Kask, Anna Markina, Zuzana Podana

**Affiliations:** ^1^Institute of Public Law, University of Tartu, Kaarli Puiestee 3, 10119 Tallinn, Estonia; ^2^Department of Sociology, Charles University, Nam. J. Palacha 2, 11638 Prague, Czech Republic

## Abstract

In Europe use of alcohol by adolescents is a large and increasing problem. The aim of this study is to examine the effects of family factors such as structure, social control, affluence, and negative life events on adolescents' risky alcohol use. Data on alcohol use and family factors were obtained from the International Self-Report Delinquency Study (ISRD-2). Using multilevel analysis, it was found that overall, complete family and high social control by parents were lowering the intense alcohol use whereas negative life events in the family and high family affluence were increasing youngsters' intense alcohol use. Differences between regions of Europe were present for all family factors except affluence. Namely, in Northern Europe the impact of family structure and social control on intense alcohol use was stronger than that in other regions (e.g., Western Europe, Mediterranean, and Postsocialist countries). Also, in Northern Europe where the proportion of adolescents who have not experienced negative life events is the highest, the impact of negative life events on intense alcohol use was stronger; that is, negative life events increased the alcohol use. We conclude that family plays a significant role in adolescents' risky alcohol use.

## 1. Introduction

Alcohol use in adolescence is strongly influenced by social and environmental factors [1]. Several studies have examined which family factors can explain delinquency in adolescents. For example, the factors which increase the risk of adolescent delinquency are related to the lack of warmth, low supervision, harsh punishment, conflictual family climate, and problems of parents within the family [2, 3].

The nature of the association between family factors and youth's intensive alcohol use is complex. Hirschi's social control theory is one of the most influential theories concerning the role of family [4]. Hirschi noted that young people who have strong bonding with their parents would interiorize the values and norms of their parents which results in behaving in a norm conforming way. According to Luthar et al. family factors can act as protective buffers against the negative effect of a high-risk context (i.e., protective enhancing effect) [5]. Schonberg and Shaw have indicated that family protective factors are influential in high-risk contexts [6]. Parental supervision in their research was found to vary in importance across contextual conditions. Cleveland et al. [7] noted that family protective factors (e.g., attachment, supervision, and discipline) offered less protection for students in high-risk school contexts (see also [8, 9]).

In this paper we are interested in which family factors are associated with intense drinking in juveniles in different regions of Europe. Thus, principles of clustering countries are first introduced followed by closer look to the effects of different family factors on alcohol use among adolescents.

### 1.1. Clustering Countries

For both practical and theoretical reasons, we sought for an empirical method to cluster the 25 countries involved in the Second International Self Report-Delinquency Study (ISRD-2). In classifying countries we used the idea of different national welfare regimes [10, 11]. Their view is based on the principle that all individuals provide for their needs by producing essentially goods and services in three different ways: (1) they work on the market place and get paid; (2) they pay taxes to the state and they may expect in return important public services and income transfers; and (3) the civil society (charities) and family offer services and support [10, 11]. Esping-Andersen [10, 12] categorized societies into three types of social organization: the social democratic model (Scandinavian countries), the liberal model (Anglo-Saxon countries), and the corporatist model (continental Europe).

The Scandinavian model is based on the idea of *equality* between citizens and is characterised by an extensive role of the state and an elaborate welfare system, reducing inequalities and fighting social exclusion which results in high taxes and extensive income transfers providing among others for free health care, free education, qualitatively good childcare and generously paid parental leave. In this social democratic model the state takes a large responsibility for its citizens' wellbeing.

The ideological basis of the Anglo-Saxon liberal model is *freedom*, expressed by the market economy which is based on the view that income has to be generated either by participation in the labour market or by family support, and people would have to cover social risks by private insurance. In this model the social security is seen as a last option, social expenditures are limited, and benefits are low and restricted in time.

The continental model considers largely *the risks* of workers during their working life (i.e., unemployment, illnesses). This model provides for income on the basis of employment history and last earned income. Specific organizations are responsible for taking care of social security benefits but the state imposes social insurance through contributions from employers and employees.

Lately, Latin or Southern model was added [13–15] along with Postsocialist model [16, 17]. The South European model is more restricted than the continental model since it is based on the principle of *subsidiarity*, in which the (extended) family is the main source of providing for social security with the church and charities forming a fall-back position in case of extreme poverty. Postsocialist model consists of the East European countries with flexible and transfer-oriented labour market.

Using the Esping-Andersen typology elaborated by Saint-Arnaid and Bernard [11], the countries were grouped into four country clusters in our study: Western Europe (Germany, France, Belgium, The Netherlands, Austria, Ireland, and Switzerland), Northern Europe (Finland, Sweden, Norway, Denmark, and Iceland), Mediterranean countries (Spain, Italy, Portugal, and Cyprus), and finally Postsocialist countries (Czech Republic, Poland, Hungary, Estonia, Lithuania, Slovenia, Bosnia-Herzegovina, Armenia, and Russia).

Apart from extending the classification scheme of Saint-Arnaid and Bernard, we differ from their classification of countries in four aspects: Iceland that originally belongs to the cluster of liberal welfare regimes is placed into the Northern European cluster, Ireland (also liberal welfare regime) is placed into the Western Europe cluster as it is the only Anglo-Saxon country in our study, Switzerland which was not a part of Saint-Arnaid's and Bernard's analyses is placed within the Western European cluster, and Cyprus which was also lacking in their analyses takes the position of Greece. Finally, we believe that the country clusters provide a useful organizing framework for analysing a large number of countries simultaneously.

### 1.2. Family Factors

*Family structure*, that is, whether the child has both parents present at home or not, has negative effects on the social behaviour of children and supervision at homes, and it is a major determinant of delinquency [18, 19]. Single parents have often fewer financial and coping resources compared to traditional two-parent families [20–22]. Also, youngsters from single-parent families are more likely to make decisions without consulting a parent [23].

Those adolescents living in one-parent households are more likely to be involved in risky use of alcohol [24]. They reported that youth living in single-parent households had higher rates of drinking alcohol compared to those living in two-parent households. Bjarnason et al. [25] noted that adolescents living with both biological parents engaged less frequently in heavy alcohol use than those living in any other arrangements. Oman et al. [26] reported that youth living in one-parent households are more likely to report using alcohol in the past thirty days. Only a few studies have found that no differences in substance use by adolescents in two-parent or single-parent families [27] or that single-mother families are no more likely to be at risk for alcohol and other drug abuse [28].

Research demonstrates that supervision and family control are strong predictors of delinquency [29, 30]. Junger-Tas et al. [19] noted that *family social control* is based on two dimensions, indirect and direct control. Indirect control is affected by the quality of the relationship of a youngster with his parents [3], whereas direct control in the family is applied by close supervision. For example, White and Halliwell [31] have found that family dinners have a positive effect on lowering the likelihood of alcohol use (see also [32]). Parental support has been associated with decreased alcohol consumption [33].

Another factor connected to youth alcohol consumption is *affluence*, that is, whether the adolescent or his/her family owns certain things (own room, PC, or car). The concept of affluence in ISRD-2 is different from socioeconomic status (SES) although there are some connecting links. Studies concerning parents' SES have found that it is positively connected with larger alcohol intake [34]. Elgar et al. [35] note that income inequality was associated with drinking frequency among 11- and 13-year-olds and drunkenness among 11-year-olds.

Some studies have identified a higher risk of excessive adolescent drinking behaviour among lower SES groups [36–38]; others have found weak effect of SES on adolescent alcohol consumption [39, 40]. Two Finnish studies found a clear relationship between adolescents' own financial resources and drunkenness [37, 41]. *Negative life events* (concerning parental conflicts and alcohol abuse) experienced by adolescents during their lifetime have a large effect on their behaviour. For example, Burt et al. [42] indicated that parental divorce predicts delinquency and other externalizing behaviours during childhood and adolescence. Otten et al. [43] indicated that alcohol use of the younger children was affected by alcohol use of both parents.

Several studies have examined the effects of parents on the onset and also heavy and problematic drinking of their children. For example, greater alcohol use by parents is associated with earlier use of alcohol by adolescents [44, 45]. Parental problematic alcohol use may disrupt normal social processes within the family, leading to increased levels of family disruption, family and marital conflict, financial strain, family alcohol and drug use, inadequate parenting practices, and poorer outcomes for children [46–51]. Seljamo et al. [52] found that fathers' present heavy drinking and parental early drinking were the best predictors of their children's problematic alcohol use at the age of 15. In addition, children with a family history of alcoholism demonstrate more escalation of alcohol use [53] and more often develop alcohol disorders and dependence [54] than children without a family history of alcoholic parents.

Thus, in the current paper we hypothesize that affluence and negative life events are related to more intensive use of alcohol whereas family structure and social control to less intense alcohol use among adolescents. Concerning country clusters it is hypothesized that in regions where the family structure is more complete and where social control is higher, the adolescents use alcohol less intensively. However, in regions where the affluence is higher and adolescents have experienced more negative life events, the adolescents use alcohol more intensively.

## 2. Method

### 2.1. Participants

This study involved 57,771 youngsters who participated in the Second International Self-Report Delinquency Study (ISRD-2) in 2006. In ISRD-2 data about adolescents' family, school, peers, neighbourhood, delinquency, victimization, self-control, and alcohol and drugs use was collected. For this paper we performed a secondary analysis on this data focusing only on the effects of family factors on risky alcohol use.

### 2.2. Characteristics of Alcohol Use

The mean age of first time use of beer/wine is smaller (*M* = 11.54, SD = 2.33) than the age for strong alcohol (*M* = 12.69, SD = 1.86; see Table 1). By country clusters, the age of first time drinking beer/wine is the smallest in Postsocialist countries (*M* = 11.12, SD = 2.51), larger in Mediterranean countries (*M* = 11.37, SD = 2.19) and Western Europe (*M* = 11.94, SD = 2.07), and the largest in Northern Europe (*M* = 12.15, SD = 2.19). The age of first time use of strong alcohol is the smallest again in Postsocialist countries (*M* = 12.33, SD = 2.05), larger in Mediterranean countries (*M* = 12.57, SD = 1.72) and Northern Europe (*M* = 12.95, SD = 1.80), and the largest in Western Europe (*M* = 13.06, SD = 1.63).

### 2.3. Dependent Variable

Our outcome variable was intense alcohol use. This variable is a dichotomous variable coded as “1” if an adolescent has an intense drinking pattern and “0” if not. Intense users are defined as follows: if the adolescent has drunk alcohol during the last month and is younger than 14 years or the adolescent has drunk alcohol during the last month, is older than 14 years, and drinks more than five times during the last 30 days or more than five units of alcohol at last drinking occasion. This variable was used in our analyses as binge drinking (e.g., drinking at least five drinks in a row) does not take into account the age factor—in younger adolescents alcohol drinking can be considered problematic even when consumed regularly but in smaller amounts.

Overall, 15.9% adolescents can be classified as intense alcohol users. When we examine country clusters, then in Western Europe the proportion of intense alcohol users is the highest (18.2%), in Postsocialist countries and Northern Europe the proportion is lower (15.3% and 14.6%, resp.), and in Mediterranean countries the lowest (12.7%). By countries, in Denmark, The Netherlands, and Czech Republic the proportion of intense alcohol users is the highest whereas in Bosnia-Herzegovina, Iceland, France, and Portugal the proportion is the lowest (see Table 1).

### 2.4. Independent Variables

For the individual-level analysis the variables were family structure, bonding, affluence, parental supervision, and negative life events.

Concerning *family structure* 25.1% of the sample lived with single-parent or step-parent households and 74.9% lived with both parents at home. For the analysis the sample living with single-parent or step-parent households was coded as “0” and those living with both parents at home coded as “1.” When we examine differences in country clusters, then in Mediterranean countries 84.1% (*n* = 7560) of adolescents live in complete family, in Postsocialist countries the proportion is 76.4% (*n* = 19848), in Western Europe 73% (*n* = 17723), and in Northern Europe 65.4% (*n* = 11934). When different countries are examined, then in Armenia and Cyprus almost 90% of adolescents live with both parents, whereas in Estonia, Finland, and Sweden the proportion is the smallest (see Table 1).

*Family bonding* is a combined variable which consists of four variables, that is, whether the adolescent gets along with father (from 1 “not at all” to 4 “very well”), gets along with mother (from 1 “not at all” to 4 “very well”), spends leisure time together with parents (from 1 “never” to 6 “more than once a week”), and has dinner with his/her family (from 1 “never” to 8 “daily”). In the analysis family bonding was standardized. When we observe differences in country clusters, then in Mediterranean countries 20.3% (*n* = 7565) of adolescents reported maximum bonding (getting very well along with father and mother, spending leisure time together with parents more than once a week, and having daily dinner with the family). In other regions this proportion was lower, namely, 14.9% (*n* = 19660) in Postsocialist countries, 14.9% (*n* = 17674) in Western Europe, and 13.6% (*n* = 11906) in Northern Europe. As per countries, in Cyprus and Armenia bonding is the strongest, whereas in Estonia, Czech Republic, and Finland it is the weakest (see Table 1).

Concerning *parental supervision* 5.4% of the sample indicated that they get supervised rarely or never, 35.3% sometimes, and 59.3% always (or they do not go out). In the analysis parental supervision was standardized. When we examine differences in country clusters, then parental supervision was most prevalent in Mediterranean countries (73.3%, *n* = 7520), followed by Western Europe (57.7%, *n* = 17446), Postsocialist countries (55.2%, *n* = 19773), and Northern Europe (52.9%, *n* = 11951). Parental supervision is the strongest in Spain, Armenia, and Bosnia-Herzegovina, whereas in Estonia, Czech Republic, and Iceland it is the lowest (see Table 1).

*Family affluence* is a combined variable which consists of four variables; that is, whether the adolescent has his/her own room (yes or no); his/her own PC (yes or no); his/her own mobile phone (yes or no), and if the family has a car (yes or no). In the analysis family affluence was standardized. When we observe differences in country clusters, then the proportion of those adolescents who answered “yes” to all the questions about owning their own room, PC, and mobile phone and parents owning a car was the highest in Northern Europe (80%, *n* = 12055), followed by Western Europe (64.4%, *n* = 17761), Mediterranean countries (59%, *n* = 7565), and Postsocialist countries (49.9%, *n* = 19892). When different countries are observed, then the proportion was the highest in Iceland and Norway and the lowest in Armenia and Russia (see Table 1).

*Negative life events* concerning family disruption consist of three variables, that is, whether the adolescent has experienced parents' use of alcohol and/or drugs, violence of parents, and parents' separation or divorce. In the analysis negative life events were standardized. In Mediterranean countries the proportion of those adolescents who have experienced any of the negative life events was the highest (81.0%, *n* = 7459); in Postsocialist countries and Western Europe this proportion was lower (74.3%, *n* = 19439 and 69.6%, *n* = 17525, resp.); and in Northern Europe this proportion was the lowest (62.9%, *n* = 11949). In Denmark and Sweden the proportion of those who have not experienced any negative life events was the lowest whereas in Armenia and Bosnia-Herzegovina it was the highest (see Table 1).

### 2.5. Statistical Analysis

In this study multilevel logistic regression analysis was used to estimate the effects of family factors on juveniles' intense drinking. The analysis was conducted in R 2.15.0; the package lme4 was used for doing all the analyses. Laplace approximation was used to estimate the parameters in the models. The first level of the multilevel analysis is the individual level of the youngsters concerning intense alcohol use. These youngsters were clustered within schools (second level). The third level of the analysis is the country level. Explanatory variables included social demographic variables (grade, gender, and migrant status) and five family factors (family structure, affluence, bonding, negative life events, and parental supervision).

The analyses of the effect of family factors on alcohol use were controlled for gender, grade, and immigrant status. Concerning gender, females were coded as “0” and males as “1.” Regarding immigrant status, the youngsters were divided into two groups: natives (coded as “1”) and 1st/2nd generation migrants (coded as “0”). Finally, grade was entered as two dummy variables in the model (the youngsters in the 7th grade were used as a reference group which were compared separately against the 8th and the 9th graders).

## 3. Results

### 3.1. Family Structure

Results for family structure are presented in Table 2. In Model 0 we see that the odds ratio of being an intense alcohol user is .16. In Model 1 we added the sociodemographic variables (gender, grade, and migrant status). The table indicates that boys are more likely to consume alcohol more intensively than girls (OR = 1.43). Youngsters with a migrant status (first or second generation) were less likely to have consumed alcohol compared to the natives (OR = 1.37). Finally, adolescents in the 8th grade are less (OR = 0.91) and the 9th grade (OR = 1.09) are more likely to have been involved in risky drinking than the seventh grade students. In Model 2 we added family structure to the model. When adolescents were living with both parents, then it lowered the likelihood of intense alcohol use (OR = .72) compared to single-parent households. The model including family structure fits better than Model 1 (*χ* ^2^(1) = 136, *P* < .001).

In Model 3 random slope variance on the country level is estimated for the impact of family structure. This random slope variance was found to be significant (*χ* ^2^(2) = 9, *P* < .01), meaning that there are differences in the impact of family structure on intense alcohol use also across countries (see Table 3). The correlation between the intercepts and slopes of the countries is negative (−.554), which indicates that the higher the intercept, the smaller the impact of family structure on the intense alcohol use in a country.

In Models 4 and 5 we added country clusters to the analysis. Model 4 was found to be not significantly better than Model 3 (*χ* ^2^(3) = 4.3, ns), although there was a difference between Northern and Western Europe on intense alcohol use. Model 5 was found to be significantly better than Model 3 (*χ* ^2^(6) = 27.8, *P* < .001) and there were differences present between clusters in the impact of family structure on intense alcohol use. Namely, in Northern Europe the impact of family structure on intense alcohol use was strongest (i.e., complete family much lowers the alcohol use compared to incomplete family) compared to Western Europe, Mediterranean, and Postsocialist countries (where the effect of complete family on the alcohol use was the weakest).

### 3.2. Family Social Control

First the results concerning bonding (see Table 4) and then parental supervision (see Table 5) are discussed. Models 0 and 1 are similar to those described in detail in the previous section; therefore for this and following factors these models are not overviewed. In Model 2 we added bonding to the model. When the bonding to the parents is higher it lowers the presence of intense alcohol drinking (OR = .68). The model including bonding fits better than Model 1 (*χ* ^2^(1) = 1020, *P* < .001).

In Model 3 we examined the interaction effect between country and bonding. There are differences in the impact of bonding across countries (see Table 3). The correlation between the intercepts and slopes of the countries is negative (−.185) which indicates that the higher the intercept, the smaller the impact of bonding on the intense alcohol use in a country. The model including bonding fits better than Model 2 (*χ* ^2^(2) = 48, *P* < .001).

In Models 4 and 5 we added country clusters to the analysis. Model 4 was found to be not significantly better than Model 3 (*χ* ^2^(3) = 5.2, ns). Model 5 was found to be significantly better than Model 3 (*χ* ^2^(6) = 29.0, *P* < .001) and there were differences present between clusters in the impact of bonding on intense alcohol use. Namely, in Northern Europe the impact of bonding on intense alcohol use was the strongest (i.e., strong bonding lowered the alcohol use) compared to Western Europe, Postsocialist countries, and Mediterranean countries (where the effect of bonding on the alcohol use was the weakest).

Now the results on parental supervision are examined. In Model 2 we added parental supervision to the model which lowered the likelihood of intense alcohol use (OR = 0.62). The model including parental supervision fits better than Model 1 (*χ* ^2^(1) = 1592, *P* < .001). In Model 3 we examined the interaction effect between country and parental supervision. There was significant association present which indicates that there are differences in the impact of supervision across countries (see Table 3). The correlation between the intercepts and slopes of the countries is positive (.204), which indicates that the higher the intercept, the higher the impact of supervision in a country. The model including supervision fits better than model 2 (*χ* ^2^(2) = 20, *P* < .001).

In Models 4 and 5 we added country clusters to the analysis. Model 4 was found to be not significantly better than Model 3 (*χ* ^2^(3) = 4.6, ns). Model 5 was found to be significantly better than Model 3 (*χ* ^2^(6) = 13.5, *P* < .05) and there were differences present between clusters in the impact of parental supervision on intense alcohol use. Namely, in Northern Europe the impact of parental supervision on intense alcohol use was the strongest (i.e., strong parental supervision lowered the alcohol use) compared to Postsocialist and Mediterranean countries and Western Europe (where the effect of parental supervision on the alcohol use was the weakest).

### 3.3. Affluence

Next, family affluence is examined (see Table 6). In Model 2 we added affluence to the model which increased the odds of intense alcohol use by 17% (OR = 1.17). It means that those adolescents who are more affluent in our terms have significantly higher odds of intense drinking. The model fits better than Model 1 (*χ* ^2^(1) = 103, *P* < .001). In Model 3 we examined the interaction effect between country and affluence. There are differences in the impact of affluence across countries (see Table 3). The correlation between the intercepts and slopes of the countries is slightly positive (.025), which indicates that the higher the intercept, the higher the impact of affluence on a country. The model fits better than Model 2 (*χ* ^2^(2) = 26, *P* < .001).

In Models 4 and 5 we added country clusters to the analysis. Model 4 was found to be not significantly better than Model 3 (*χ* ^2^(3) = 5.4, ns). Model 5 was found to be significantly better than Model 3 (*χ* ^2^(6) = 12.8, *P* < .05); however, there were also no differences between clusters in the impact of affluence on intense alcohol use.

### 3.4. Negative Life Events

Finally, negative life events are studied (see Table 7). In Model 2 we added negative life events to the model. The increase in negative life events increased the odds of intense alcohol use by 25% (OR = 1.25). The model fits better than Model 1 (*χ* ^2^(1) = 358, *P* < .001). In Model 3 we examine the interaction effect between country and negative life events. It was again confirmed that there are differences in the impact of negative life events across countries (see Table 3). The correlation between the intercepts and slopes of the countries is positive (.128). The model fits better than Model 2 (*χ* ^2^(2) = 2, *P* < .01).

In Models 4 and 5 we added country clusters to the analysis. Model 4 was found to be not significantly better than Model 3 (*χ* ^2^(3) = 5.9, ns). Model 5 was found to be significantly better than Model 3 (*χ* ^2^(6) = 34.2, *P* < .001), and there were differences present between clusters in the impact of negative life events on intense alcohol use. Namely, in Northern Europe the impact of negative life events on intense alcohol use was the strongest (i.e., negative life events increased the alcohol use) compared to Western Europe, Mediterranean, and Postsocialist countries (where the effect of negative life events on the alcohol use was the weakest).

## 4. Discussion

In this chapter we examined the intense alcohol use of youngsters from 25 European countries and to what extent the intense alcohol use was associated with different family factors, that is, structure, bonding, supervision, affluence, and negative life events, and also the differences between country clusters were observed. The analysis was controlled for similar background information, that is, gender, grade, and migrant status.

Concerning family structure our hypotheses were confirmed. The adolescents who came from two-parent households were less likely intense alcohol users than those from single-parent households which confirms some previous findings [24, 26, 35]. Multilevel analysis demonstrated that there were differences between countries and also between country clusters. Namely, in Northern Europe the impact of family structure on intense alcohol use was stronger than in other regions (Western Europe, Mediterranean, and Postsocialist countries). Thus, on one hand, in Northern Europe the proportion of adolescents who live with both parents was the smallest, yet the effect of living with both parents on intense alcohol use was the strongest. Interestingly, in Mediterranean and Postsocialist countries the impact of family structure on intense alcohol use was similar; however, in Mediterranean countries the proportion of both parents living with the adolescents is higher.

Regarding social control, the adolescents in families with stronger bonding and parental supervision were less involved with intense alcohol use which supports previous research [31–33]. The differences in intensive alcohol use emerged between countries and also between country clusters. In Northern Europe the impact of bonding and parental supervision on intense alcohol use was the strongest compared to other regions (i.e., strong bonding and parental control lowered the intense alcohol use). Interestingly, strong bonding was most prevalent in Mediterranean countries; however, the impact of bonding on intense alcohol use was there the weakest (similar results but in a lesser extent emerged for parental supervision). One possible reason for this finding could be that in Mediterranean countries the proportion of intense alcohol users is the lowest compared to other regions.

Although the scale of affluence has been used already in international research [55], the analysis of ISRD-2 data questions the value of this scale as a measure of affluence. Marshall and Enzmann have proposed that in affluent societies the scale measures the propensity to consume instead [56]. As this scale was the best available indicator for SES, it was included into analysis; however, the results shall be interpreted with care. Affluence was found to be related to intense alcohol use; that is, when adolescents were from more affluent family, then it increased the intense alcohol use which confirms previous findings [34, 37, 41]. There were differences in affluence between different countries; that is, in some countries affluent youngsters were more intense alcohol users than in other countries which confirms Marshall's and Enzmann's hypothesis [56]. However, there were no differences between country clusters in the impact of affluence on intense alcohol use. This finding is interesting because the differences in affluence were relatively large, from 49.9% in Postsocialist countries to 80% in Northern Europe.

Last, we found support for the hypothesis that when adolescents experience more negative life events, then it will increase their intense drinking (see also [43, 53]). There were differences in the association of negative life events with intense alcohol use between countries, and also the differences were present between country clusters. In Northern Europe, where the proportion of adolescents who have experienced more negative life events is the highest, the impact of negative life events on intense alcohol use was stronger than in other regions (i.e., negative life events increased the alcohol use). One important factor influencing this can be the divorce rate which is relatively low in Mediterranean and Postsocialist countries compared to Northern Europe.

Overall, we can conclude that all family factors were highly related to intense alcohol use among adolescents which confirms Hirschi's social control theory [4]. Family structure and social control were lowering the intense alcohol use, whereas negative life events and affluence were increasing youngsters' intense alcohol use. Differences between country clusters were present for all family factors except affluence.

## Figures and Tables

**Table 1:** Mean ages of onset of using alcohol and proportion of intense alcohol users and family factors in different countries (%).

Country	Age of onset of beer/wine	Age of onset of strong alcohol	Intense alcohol use	Structure	Bonding	Parental supervision	Affluence	Negative life events
	*M* (SD)	*M* (SD)	%	%	%	%	%	%
Armenia	11.16 (2.53)	11.85 (2.47)	9	89.8	30.5	74.7	20	93.5
Austria	11.92 (2.15)	13.09 (1.55)	20.3	71.6	15.1	57.9	72.3	67.6
Belgium	11.70 (2.06)	12.79 (1.81)	18.6	68.3	16.7	59.6	52	63.2
Bosnia and Herzegovina	11.39 (2.42)	11.91 (2.26)	5.4	83.1	25.2	74.2	38.8	90
Cyprus	10.91 (2.25)	11.78 (2.06)	14	89.6	36.3	72.9	62.8	80.9
Czech Republic	10.44 (2.55)	11.99 (1.97)	23.6	70.3	6.2	43.9	54.8	67.5
Denmark	12.12 (1.90)	12.46 (1.89)	25.5	65.5	14.3	56.4	77.3	54.1
Estonia	11.01 (2.58)	12.37 (2.07)	20.6	62	4.7	34.4	57.5	61.8
Finland	12.22 (2.18)	13.40 (1.48)	18.9	62.2	6.6	50.4	73.2	60.3
France	11.35 (2.21)	12.53 (1.79)	7.6	67.6	18.3	57.4	45.2	66.8
Germany	12.08 (1.98)	13.18 (1.46)	23.5	71.4	12.4	56.4	75.6	66.3
Hungary	11.73 (2.29)	12.93 (1.70)	15	75	12	52.6	58.2	66.8
Iceland	11.19 (2.28)	12.11 (.91)	7.5	70.6	21.3	44.5	88.8	73.6
Ireland	12.14 (1.98)	12.85 (1.61)	22	80.8	12.2	49	65.2	79.3
Italy	11.52 (2.29)	13.19 (1.65)	15.6	83.9	19.9	71.3	53.8	80.3
Lithuania	10.98 (2.50)	12.24 (2.21)	18.7	74.9	12.7	49.5	53.2	67.8
Netherlands	11.60 (1.93)	12.90 (1.73)	23.6	75.1	16.7	63.3	77.9	72.7
Norway	12.55 (2.13)	13.45 (1.65)	10.8	66.4	14.6	56.1	82.8	67.2
Poland	12.28 (2.26)	13.16 (1.87)	12.9	82.1	12.9	54.9	54.7	75.1
Portugal	11.68 (2.16)	12.55 (1.65)	7.9	79.7	6.5	73	59.4	80.9
Russia	11.71 (2.26)	12.68 (1.96)	8.8	70.6	12.5	52.8	29.7	68.7
Slovenia	10.63 (2.58)	12.07 (2.01)	18	79.7	16.3	58.4	73.4	76.5
Spain	12.97 (1.67)	13.56 (1.42)	10	81.8	15.2	78.1	58.3	82.5
Sweden	11.94 (2.39)	12.83 (1.91)	10.5	62.1	11.4	57.3	77.6	59.3
Switzerland	12.27 (2.11)	13.38 (1.58)	17.8	76.2	13.6	62.3	75.2	68.3

**Table 2:** The results of multilevel analysis concerning family structure (*n* individuals: 53053; *n* schools: 1344; *n* countries: 25).

	Model 0: empty model	Model 1: control variables	Model 2: family structure	Model 3: family structure random slope	Model 4: country cluster	Model 5: family structure × country cluster
Fixed	exp(*B*) (S.E.)	exp(*B*) (S.E.)	exp(*B*) (S.E.)	exp(*B*) (S.E.)	exp(*B*) (S.E.)	exp(*B*) (S.E.)
Intercept	.16 (.02)***	.10 (.01)***	.13 (.01)***	.13 (.01)***	.10 (.02)***	.14 (.02)***
Grade 8 (ref. grade 7)		.91 (.03)***	.91 (.03)***	.91 (.03)***	.91 (.03)**	.91 (.07)**
Grade 9 (ref. grade 7)		1.09 (.04)*	1.09 (.04)*	1.09 (.04)*	1.09 (.04)*	1.09 (.03)*
Male (ref. female)		1.43 (.04)***	1.45 (.04)***	1.45 (.04)***	1.45 (.04)***	1.45 (.03)***
Native (ref. migrant)		1.37 (.05)***	1.37 (.05)***	1.37 (.05)***	1.37 (.05)***	1.37 (.03)***
Family structure (ref. incomplete family)			.72 (.02)***	.73 (.03)***	.73 (.03)***	.55 (.11)***
Western Europe (ref. Northern Europe, NE)					1.68 (.44)*	1.34 (.29)
Mediterranean countries (ref. NE)					1.10 (.33)	.63 (.36)
Postsocialist countries (ref. NE)					1.36 (.34)	.75 (.27)
Family structure × Western Europe (ref. NE)						1.22 (.03)*
Family structure × Mediterranean countries (ref. NE)						1.52 (.01)***
Family structure × Postsocialist countries (ref. NE)						1.56 (.01)***
Random						
Var. school	.273	.254	.253	.254	.254	.255
Var. country	.246	.240	.234	.293	.273	.205
Var. family structure				.024	.026	.001
Cor. family structure, intercept				−.554	−.645	−1.000
LR test	*χ* ^2^(2) = 1850***	*χ* ^2^(4) = 317***	*χ* ^2^(1) = 136***	*χ* ^2^(2) = 9**	*χ* ^2^(3)= 4.3 ns	*χ* ^2^(6) = 27.8***

***P < .001; **P < .01; *P < .05. Models 4 and 5 are compared to Model 3.

**Table 3:** Adjusted odds ratios for five family variables by country (controlled for gender, grade, and migrant status).

Country	Family structure	Family bonding	Parental supervision	Affluence	Negative life events
Armenia	1.95	0.90	0.72	1.18	1.08
Austria	0.63	0.64	0.68	1.24	1.25
Belgium	0.68	0.70	0.65	1.25	1.21
Bosnia and Herzegovina	1.51	0.66	0.58	1.19	1.18
Cyprus	0.95	0.74	0.52	1.12	1.30
Czech Republic	0.88	0.80	0.64	1.12	1.10
Denmark	0.54	0.57	0.57	1.17	1.31
Estonia	0.85	0.72	0.61	1.11	1.19
Finland	0.64	0.54	0.46	1.18	1.37
France	0.63	0.68	0.71	1.27	1.26
Germany	0.67	0.66	0.66	1.76	1.29
Hungary	0.79	0.65	0.59	1.10	1.06
Iceland	0.35	0.55	0.47	0.58	1.74
Ireland	0.55	0.62	0.52	0.83	1.45
Italy	0.76	0.73	0.66	1.18	1.20
Lithuania	0.72	0.77	0.61	1.02	1.16
Netherlands	0.79	0.68	0.70	1.58	1.31
Norway	0.67	0.64	0.61	1.20	1.31
Poland	0.71	0.74	0.54	1.10	1.21
Portugal	0.92	1.00	0.66	1.17	1.09
Russia	0.77	0.59	0.57	1.24	1.17
Slovenia	0.72	0.68	0.54	0.91	1.19
Spain	0.87	0.79	0.69	1.21	1.26
Sweden	0.51	0.65	0.51	1.21	1.42
Switzerland	0.75	0.59	0.59	1.27	1.32

**Table 4:** The results of multilevel analysis concerning bonding (last model: *n* individuals 52724; *n* schools 1344; *n* countries: 25).

	Model 0: empty model	Model 1: control variables	Model 2: bonding	Model 3: cross-level interaction bonding random slope	Model 4: country cluster	Model 5: family bonding × country cluster
Fixed	exp(*B*) (S.E.)	exp(*B*) (S.E.)	exp(*B*) (S.E.)	exp(*B*) (S.E.)	exp(*B*) (S.E.)	exp(*B*) (S.E.)
Intercept	.16 (.02)***	.10 (.01)***	.10 (.01)***	.10 (.01)***	.10 (.02)***	.09 (.02)***
Grade 8 (ref. grade 7)		.91 (.03)***	.86 (.03)***	.86 (.03)***	.86 (.03)***	.85 (.07)***
Grade 9 (ref. grade 7)		1.09 (.04)**	.96 (.03) ns	.96 (.03) ns	.95 (.03)	.96 (.03) ns
Male (ref. female)		1.43 (.04)***	1.51 (.04)***	1.51 (.04)***	1.51 (.04)***	1.51 (.03)***
Native (ref. migrant)		1.37 (.05)***	1.43 (.05)***	1.43 (.05)***	1.43 (.05)***	1.44 (.04)***
bonding			.68 (.01)***	.68 (.02)***	.68 (.02)***	.57 (.06)***
Western Europe (ref. Northern Europe, NE)					1.40 (.35)	1.49 (.27)
Mediterranean countries (ref. NE)					.69 (.20)	.84 (.32)
Postsocialist countries (ref. NE)					.82 (.20)	.96 (.25)
bonding x Western Europe (ref. NE)						1.12 (.02)*
bonding x Mediterranean countries (ref. NE)						1.37 (.01)***
bonding x Postsocialist countries (ref. NE)						1.26 (.01)***
Random						
Var. school	.271	.252	.252	.249	.250	.250
Var. country	.244	.239	.223	.224	.182	.177
Var. bonding				.013	.013	.002
Cor. bonding, intercept				−.185	.227	.167
LR test	*χ* ^2^(2) = 1830***	*χ* ^2^(4) = 315***	*χ* ^2^(1) = 1020***	*χ* ^2^(2) = 48***	*χ* ^2^(3) = 5.2 ns	*χ* ^2^(6) = 29***

***P < .001; **P < .01; *P < .05. Models 4 and 5 are compared to Model 3.

**Table 5:** The results of multilevel analysis concerning parental supervision (*n* individuals: 52564; *n* schools: 1344; *n* countries: 25).

	Model 0: empty model	Model 1: control variables	Model 2: parental supervision	Model 3: parental supervision random slope	Model 4: country cluster	Model 5: parental supervision × country cluster
Fixed	exp(*B*) (S.E.)	exp(*B*) (S.E.)	exp(*B*) (S.E.)	exp(*B*) (S.E.)	exp(*B*) (S.E.)	exp(*B*) (S.E.)
Intercept	.16 (.02)***	.10 (.01)***	.10 (.01)***	.10 (.01)***	.09 (.02)***	.09 (.02)***
Grade 8 (ref. grade 7)		.90 (.03)***	.85 (.03)***	.86 (.03)***	.86 (.03)***	.86 (.07)***
Grade 9 (ref. grade 7)		1.09 (.04)**	1.02 (.03) ns	1.01 (.03) ns	1.01 (.04) ns	1.01 (.03) ns
Male (ref. female)		1.43 (.04)***	1.29 (.03)***	1.29 (.03)***	1.29 (.03)***	1.29 (.03)***
Native (ref. migrant)		1.35 (.05)***	1.46 (.05)***	1.46 (.05)***	1.46 (.05)***	1.46 (.04)***
Parental supervision			.62 (.01)***	.61 (.01)***	.61 (.01)***	.53 (.07)***
Western Europe (ref. Northern Europe, NE)					1.52 (.39)	1.59 (.27)
Mediterranean countries (ref. NE)					.93 (.27)	.97 (.33)
Postsocialist countries (ref. NE)					.95 (.23)	.99 (.25)
Parental supervision × Western Europe (ref. NE)						1.20 (.02)**
Parental supervision × Mediterranean countries (ref. NE)						1.17 (.01)*
Parental supervision × Postsocialist countries (ref. NE)						1.14 (.01)*
Random						
Var. school	.277	.258	.258	.257	.257	.257
Var. country	.248	.242	.220	.225	.178	.181
Var parental supervision				.007	.007	.004
Cor parental supervision, intercept				.204	.019	.083
LR test	*χ* ^2^(2) = 1868***	*χ* ^2^(4) = 313***	*χ* ^2^(1) = 1592***	*χ* ^2^(2) = 20***	*χ* ^2^(3) = 4.6 ns	*χ* ^2^(6) = 13.5*

***P < .001; **P < .01; *P < .05. Models 4 and 5 are compared to Model 3.

**Table 6:** The results of multilevel analysis concerning affluence (*n* individuals: 53136; *n* schools: 1344; *n* countries: 25).

	Model 0: empty model	Model 1: control variables	Model 2: affluence	Model 3: affluence random slope	Model 4: country cluster	Model 5: affluence × country cluster
Fixed	exp(*B*) (S.E.)	exp(*B*) (S.E.)	exp(*B*) (S.E.)	exp(*B*) (S.E.)	exp(*B*) (S.E.)	exp(*B*) (S.E.)
Intercept	.16 (.02)***	.10 (.01)***	.10 (.01)***	.10 (.01)***	.10 (.02)***	.10 (.02)***
Grade 8 (ref. grade 7)		.91 (.03)***	.90 (.03)***	.90 (.03)***	.90 (.03)***	.90 (.07)***
Grade 9 (ref. grade 7)		1.09 (.04)**	1.07 (.04)*	1.07 (.04)*	1.06 (.04)	1.06 (.03)
Male (ref. female)		1.43 (.04)***	1.42 (.04)***	1.42 (.04)***	1.43 (.04)***	1.43 (.03)***
Native (ref. migrant)		1.37 (.05)***	1.32 (.05)***	1.32 (.05)***	1.31 (.05)***	1.30 (.04)***
Affluence			1.17 (.02) ***	1.18 (.04) ***	1.18 (.04)***	1.19 (.12)*
Western Europe (ref. Northern Europe, NE)					1.54 (.38)	1.45 (.26)
Mediterranean countries (ref. NE)					.82 (.23)	.82 (.32)
Postsocialist countries (ref. NE)					1.02 (.24)	1.07 (.24)
Affluence × Western Europe (ref. NE)						1.10 (.03)
Affluence × Mediterranean countries (ref. NE)						.99 (.01)
Affluence × Postsocialist countries (ref. NE)						.92 (.01)
Random						
Var. school	.274	.256	.251	.245	.246	.245
Var. country	.246	.240	.216	.208	.172	.171
Var. affluence				.015	.015	.009
Cor. affluence, intercept				.025	−.245	−.233
LR test	*χ* ^2^(2) = 1859***	*χ* ^2^(4) = 318 ***	*χ* ^2^(1) = 103***	*χ* ^2^(2) = 26***	*χ* ^2^(3) = 5.4 ns	*χ* ^2^(6) = 12.8*

***P < .001; **P < .01; *P < .05. Models 4 and 5 are compared to Model 3.

**Table 7:** The results of multilevel analysis concerning negative life events (*n* individuals: 52386; *n* schools: 1344; *n* countries: 25).

	Model 0: empty model	Model 1: control variables	Model 2: negative life events	Model 3: negative life events random slope	Model 4: country cluster	Model 5: negative life events × country cluster
Fixed	exp(*B*) (S.E.)	exp(*B*) (S.E.)	exp(*B*) (S.E.)	exp(*B*) (S.E.)	exp(*B*) (S.E.)	exp(*B*) (S.E.)
Intercept	.16 (.02)***	.10 (.01)***	.10 (.01)***	.10 (.01)***	.11 (.02)***	.09 (.02)***
Grade 8 (ref. grade 7)		.91 (.03)***	.90 (.03)***	.90 (.03)***	.90 (.03)***	.90 (.07)***
Grade 9 (ref. grade 7)		1.09 (.04)**	1.07 (.04)*	1.07 (.04)*	1.07 (.04)	1.07 (.03)
Male (ref. female)		1.42 (.04)***	1.48 (.04)***	1.48 (.04)***	1.48 (.04)***	1.48 (.03)***
Native (ref. migrant)		1.36 (.05)***	1.38 (.05)***	1.38 (.05)***	1.38 (.05)***	1.38 (.04)***
Negative life events			1.25 (.02)***	1.25 (.02)***	1.26 (.02)***	1.39 (.04)***
Western Europe (ref. Northern Europe, NE)					1.33 (.32)	1.55 (.27)
Mediterranean countries (ref. NE)					.63 (.18)	.89 (.32)
Postsocialist countries (ref. NE)					.67 (.16)	1.06 (.25)
Negative life events × Western Europe (ref. NE)						.93 (.01)*
Negative life events × Mediterranean countries (ref. NE)						.86 (.01)***
Negative life events × Postsocialist countries (ref. NE)						.82 (.01)***
Random						
Var. school	.273	.254	.251	.251	.251	.251
Var. country	.248	.242	.220	.221	.210	.179
Var. negative life events				.004	.004	.001
Cor. negative life events, intercept				.128	−.637	−1.000
LR test	*χ* ^2^(2) = 1833***	*χ* ^2^(4) = 305***	*χ* ^2^(1) = 358***	*χ* ^2^(2) = 11**	*χ* ^2^(3) = 5.9 ns	*χ* ^2^(6) = 34.2***

***P < .001; **P < .01; *P < .05. Models 4 and 5 are compared to Model 3.
